# Effectiveness of the GRACE risk score according to troponin elevation in patients admitted with non-ST elevation acute coronary syndrome: a post hoc analysis of the UKGRIS parallel group cluster randomised controlled trial

**DOI:** 10.1136/openhrt-2025-003213

**Published:** 2025-06-26

**Authors:** Chris P Gale, Deborah Stocken, Ramesh Nadarajah, Suleman Aktaa, Catherine Reynolds, Rachael Gilberts, David B Brieger, Kathryn Carruthers, Derek P Chew, Shaun Goodman, Catherine Fernandez, Linda Sharples, Andrew T Yan, Keith A A Fox

**Affiliations:** 1 Leeds Institute of Cardiovascular and Metabolic Medicine, University of Leeds, Leeds, UK; 2 Leeds Institute of Data Analytics, University of Leeds, Leeds, UK; 3 Department of Cardiology, Leeds Teaching Hospitals NHS Trust, Leeds, UK; 4 Leeds Institute of Clinical Trials Research, University of Leeds, Leeds, UK; 5 University of Leeds Clinical Trials Research Unit, Leeds, UK; 6 Cardiology, Concord Hospital, Sydney, New South Wales, Australia; 7 University of Edinburgh Division of Clinical and Surgical Sciences, Edinburgh, UK; 8 Victorian Heart Hospital, Monash Health, Melbourne, Victoria, Australia; 9 Medicine, St Michael’s Hospital, Toronto, Ontario, Canada; 10 Medicine, University of Alberta, Edmonton, Alberta, Canada; 11 Medical Statistics, London School of Hygiene and Tropical Medicine, London, UK; 12 Department of Medicine, University of Toronto, Toronto, Ontario, Canada; 13 Royal Infirmary, Edinburgh, UK

**Keywords:** Myocardial Infarction, Coronary Angiography, Quality of Health Care

## Abstract

**Background:**

The effectiveness of risk stratification using the Global Registry of Acute Coronary Events (GRACE) Risk Score (GRS) for patients presenting to hospital with suspected non-ST elevation acute coronary syndrome (NSTEACS) according to troponin elevation is unknown.

**Methods:**

Post hoc analysis of a phase 3 parallel group cluster randomised controlled trial (UK GRACE Risk Score, UKGRIS) of adult patients presenting with suspected NSTEACS to 42 hospitals in England between 9 March 2017 and 30 December 2019, with hospitals randomised (1:1) to standard care or according to the GRS and associated guidelines. Coprimary outcome measures were use of guideline-recommended management and time to the composite of cardiovascular death, non-fatal myocardial infarction, new-onset heart failure hospitalisation or readmission for cardiovascular event at a minimum of 24 months follow-up.

**Results:**

A total of 3050 patients were randomised in UKGRIS, of whom 2602 had troponin elevation. The relative effect of GRS compared with standard care on the uptake of guideline-recommended care was greater for participants with troponin elevation compared with those without (relative OR 1.52, 95% CI 1.16 to 2.00, p<0.01). The time to the first composite event was not improved by the GRS among participants with (HR 0.89, 95% CI 0.70 to 1.14) or without troponin elevation (HR 1.14, 95% CI 0.79 to 1.64), with no interaction (relative HR 0.79, 95% CI 0.57 to 1.08, p=0.14 for interaction).

**Conclusions:**

For suspected NSTEACS, the effect of the GRS compared with standard care on uptake of recommended processes in those with elevated troponin was higher than in those without. However, this did not translate into a reduction in the composite primary or secondary outcomes at 24 months.

**Trial registration number:**

ISRCTN29731761.

**WHAT IS ALREADY KNOWN ON THIS TOPIC**

- Risk stratification using the Global Registry of Acute Coronary Events (GRACE) Risk Score (GRS) was not found to improve guideline adherence or reduce cardiovascular outcomes compared with standard care for patients with non-ST elevation acute coronary syndrome (NSTEACS) in the UK GRACE Risk Score (UKGRIS) randomised clinical trial (RCT).

**WHAT THIS STUDY ADDS**

- In this post hoc analysis of the UKGRIS RCT, GRS implementation improved guideline adherence in patients with troponin elevation compared with those without but did not reduce cardiovascular outcomes.

**HOW THIS STUDY MIGHT AFFECT RESEARCH, PRACTICE OR POLICY**

- Care processes for NSTEACS are embedded in UK clinical practice and so the role of GRS may be to quantify the risk–benefit trade-off in specific cases, rather than the majority.

## Introduction

 Troponin elevation in the context of non-ST elevation acute coronary syndrome (NSTEACS) is associated with high risk of adverse clinical events, including recurrent myocardial infarction (MI), heart failure and death.^1^ The management of patients with NSTEACS is guided by their estimated risk of future ischaemic events, with observational studies showing that failure to follow guideline recommendations is associated with excess mortality.^2^ Accordingly, risk stratification using the Global Registry of Acute Coronary Events (GRACE) Risk Score (GRS) to guide the management of patients with NSTEACS is advocated in clinical guidelines.^1 3 4^

The Australian GRACE Risk Intervention Study (AGRIS) trial, which randomised 2318 patients with either ST-segment elevation acute coronary syndrome or NSTEACS to GRS use or standard care, found that implementation of the GRS was associated with an increase in early invasive treatment, especially for NSTEACS but not other aspects of care, and the intervention was not associated with a statistically significant reduction in death or MI at 12 months.^5^ In the UK GRACE Risk Score (UKGRIS) randomised controlled trial, implementation of the GRS compared with standard care did not improve adherence to guideline-recommended management or reduce cardiovascular events at 12 months in patients admitted with suspected NSTEACS.^6^

Troponin elevation in the context of suspected NSTEACS may signify non-ST-segment elevation MI (NSTEMI),^1^ and in this population, the implementation of GRS could impact adherence to guideline-recommended management and outcomes. As part of the planned analysis for the first coprimary outcome measure, we performed subgroup analyses, of which one (elevated troponin on admission) significantly modified the effect of randomisation to GRACE in increasing uptake of care processes.^6^ Here, we report a post hoc analysis according to troponin elevation and extend the routine healthcare data outcomes capture to at least 24 months from the date of randomisation.

## Methods

### Trial design and participants

The UKGRIS trial was a parallel group, cluster randomised controlled trial in patients admitted with suspected NSTEACS evaluating the effectiveness of the GRS compared with standard care at one of 42 hospitals (38 clusters) in England. The design, baseline characteristics and primary results of the trial are published.^7^ The GRS V.2.0 was used.^8^

### Study patients

Patients admitted to hospital with suspected NSTEACS, defined as NSTEMI or unstable angina, but not STEMI, were eligible if they were aged ≥18 years, their NSTEACS was not precipitated by a clear non-cardiovascular cause, and they were not previously enrolled in the trial.

### Outcomes

The coprimary outcome measures were overall use of class I guideline recommended care processes, and time to the composite of cardiovascular death, non-fatal MI, new-onset heart failure hospital admission or readmission for cardiovascular event within 24 months. Secondary outcome measures included the total duration of hospital stay, EQ-5D-5L (5-Domain 5-Level version of the EuroQoL index) utilities, unscheduled revascularisation and the individual components of the composite endpoint over 24 months of follow-up.

Use of hospital healthcare and dates and causes of death during follow-up were collected using Hospital Episode Statistics from National Health Service Digital and the Civil Registration of Deaths Register of the Office for National Statistics. These data used International Classification of Diseases 10th revision codes.

### Statistical analyses

For the main trial, analyses followed a predefined statistical analysis plan.^9^ Eligible guideline recommended care processes were analysed as a three-level hierarchy (guideline, within patient, within hospital cluster) with adherence to each guideline (yes/no) the response in a logistic model including random intercepts for clusters and participants, and fixed effects for cluster-level minimisation factors, treatment arm and guideline identifier. The resulting estimate was the OR for receiving a guideline-directed process that a person was eligible to receive (based on their risk factors and medical history) in the population of all randomised participants, irrespective of any intercurrent events such as death before discharge. For the present paper, we repeated the analyses for both primary outcome measures twice. First, we replaced the randomised cluster effect from our analyses with an effect of having elevated troponin. Second, we performed analyses that included main effects for randomised cluster allocation, troponin elevation and the interaction between the two. We did not perform any similar analyses for the other subgroups. For time to event outcomes (the composite and its components), we used Kaplan-Meier estimators, stratified by troponin status or the four combinations of troponin status with randomised allocation. We estimated uncertainty in survivor function estimates by performing non-parametric bootstrap resampling of whole clusters and presenting 95% percentile-based intervals for the survivor function at regular intervals. Analysis of time to event outcomes used Cox proportional hazards models, adjusting for the cluster randomisation balancing variables (primary PCI capability and annual case volume as large/medium/small), troponin, randomised arm and arm-by-troponin interaction (where applicable). Cluster membership was modelled using Gamma-distributed frailties. Time-varying covariates for the cluster minimisation characteristics were fitted to all Cox models.

The duration of hospital stay over 24 months was analysed by linear regression models, adjusted for the minimisation factors with normally distributed random intercepts for hospitals. The analysis model for EQ-5D-5L utility (derived using the crosswalk algorithm) included a fixed effect for baseline EQ-5D-5L utility. All new analyses reported de novo in this publication were performed on the complete case dataset of those having data for outcome measures, troponin status and other baseline effects. As in the primary outcomes publication, participants were analysed within the arms assigned to their cluster at cluster allocation. All analyses were undertaken using SAS V.9.4.

### Patient and public involvement

The UKGRIS Oversight Committee had patient representation. We did not involve patients in the interpretation of the results of the trial or the writing of the primary outcomes manuscript.

## Results

### Hospitals and patient characteristics

**Figure 1:**
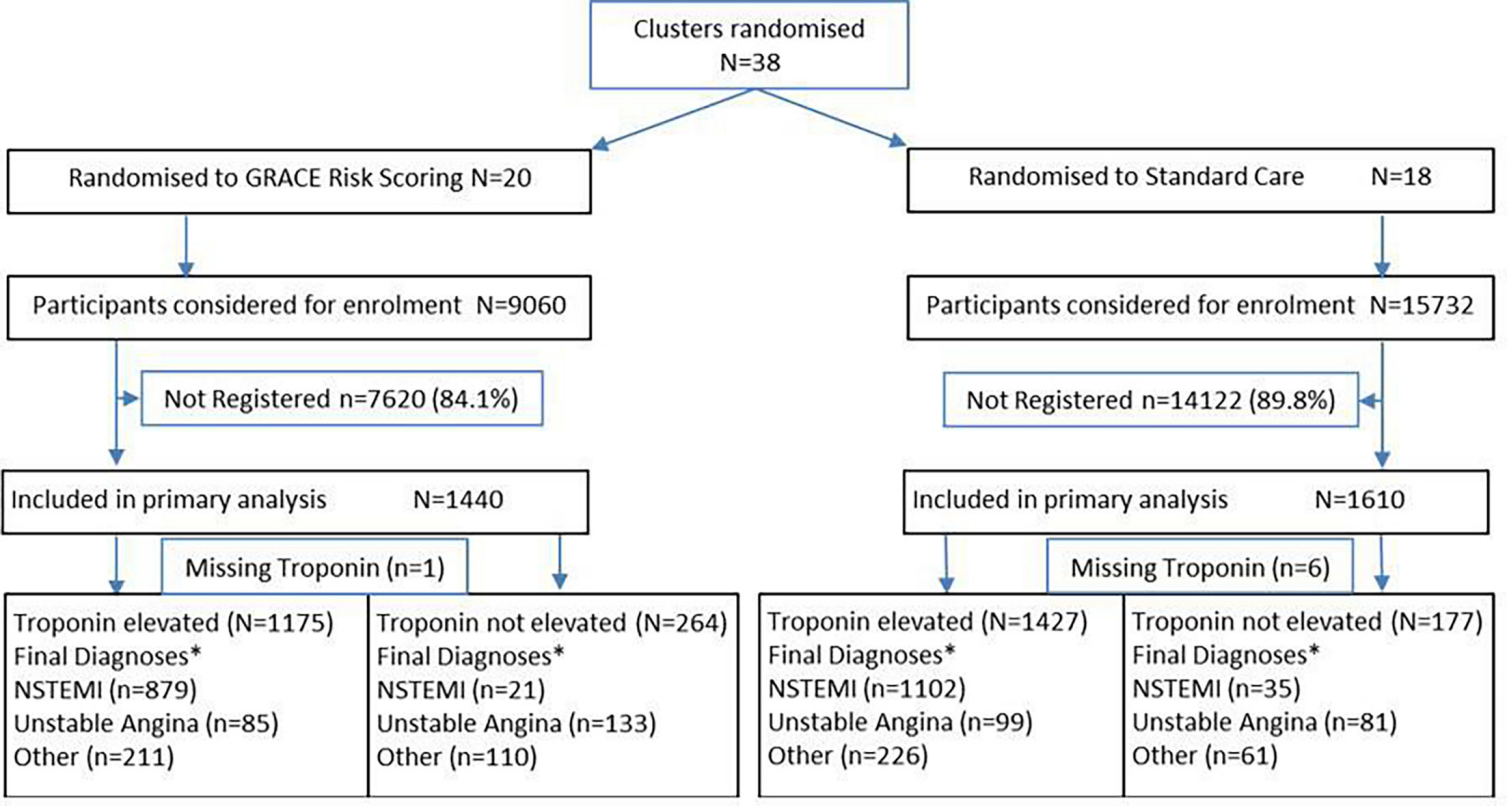
Participant recruitment and troponin status and final diagnoses. GRACE, Global Registry of Acute Coronary Events; NSTEMI, non-ST-segment elevation myocardial infarction. *As per routine records

Between 9 March 2017 and 30 December 2019, we recruited 3050 participants (1440 GRS, 1610 standard care) who were admitted with suspected NSTEACS to 42 hospitals in 38 clusters (20 GRS, 18 standard care; figure 1). Results have been published.^6^ Overall, 2602 participants had elevated troponin and 7 had missing troponin values.

Participants with troponin elevation were older, had higher GRS and were more likely to have heart failure and peripheral vascular disease, but less likely to have hypertension than those without (table 1). Both groups had similar rates of diabetes mellitus.

**Table 1:** Summaries of baseline characteristics by troponin subgroup

	Troponin elevated (n=2602)	Troponin not elevated (n=441)
Participant characteristics		
Age (years), mean (SD)	66 (12.0)	63.5 (11.8)
Male sex	1808 (69.5)	302 (68.5)
White ethnicity, mean (SD)	2364 (90.9)	385 (87.3)
Body mass index, mean (SD)	29.3 (6.0)	29.4 (5.7)
Medical history		
Hypertension	1262 (48.5)	232 (52.6)
Diabetes	695 (26.7)	116 (26.3)
Peripheral vascular disease	122 (4.7)	12 (2.7)
Congestive heart failure	135 (5.2)	17 (3.9)
Hospital admission		
ST segment deviation on ECG	756 (29.1)	85 (19.3)
Heart rate on admission (bpm), mean (SD)	76.3 (18.2)	71.6 (15.2)
Systolic blood pressure on admission (mm Hg), mean (SD)	144.0 (24.8)	140.9 (24.3)
Cardiac arrest between symptom onset and admission	11 (0.4)	0 (0.0)
Killip class		
Killip I	2354 (90.5)	415 (94.1)
Killip II	226 (8.7)	24 (5.4)
Killip III	14 (0.5)	1 (0.2)
Killip IV	5 (0.2)	0 (0.0)
Diuretics	389 (15.0)	39 (8.8)
Biomarkers		
Creatinine (mmol/L), mean (SD)	92.2 (44.6)	83.6 (25.0)
Haematocrit (%), mean (SD)	41.0 (4.9)	41.5 (4.3)
Edmonton frailty score, mean (SD)	3.6 (3.1)	4.2 (3.0)
GRACE risk score		
Mean (SD)	126.2 (32.8)	102.2 (27.6)
Categories		
Unknown	29 (1.1)	2 (0.5)
Low (≤108)	815 (31.3)	267 (60.5)
Intermediate (109–140)	929 (35.7)	130 (29.5)
High (≥141)	829 (31.9)	42 (9.5)
CRUSADE risk score		
Mean (SD)	23.7 (14.1)	20.2 (12.0)
Categories		
Unknown	81 (3.1)	17 (3.9)
Low (≤30)	1783 (68.5)	348 (78.9)
Intermediate (31–40)	415 (15.9)	47 (10.7)
High (≥41)	323 (12.4)	29 (6.6)

Data are numbers (%) unless otherwise stated.

CRUSADE=can rapid risk stratification of unstable angina patients suppress adverse outcomes with early implementation of the American College of Cardiology/American Heart Association guidelines.

GRACE, Global Registry of Acute Coronary Events.

### Processes of care

For most of the 11 guideline care processes, participants with troponin elevation at baseline who were eligible to receive such care processes had higher uptake than those eligible but without troponin elevation (table 2). Exceptions were ischaemia testing (25.7% vs 24.2% elevation vs no elevation) and invasive coronary angiography within 24 hours (12.1% vs 15.4% elevation vs no elevation).

**Table 2:** Summaries of the receipt of guideline recommended care processes by troponin subgroup

	Troponin elevated (n=2602)	Troponin not elevated (n=441)	Missing	Total
Total	15 243/19 407 (78.5)	1882/3037 (62.0)	4/29 (13.8)	17 129/22 473 (76.2)
Aspirin	2127/2198 (96.8)	348/395 (88.1)	–	2475/2593 (95.4)
Ischaemia testing	19/74 (25.7)	23/95 (24.2)	–	42/169 (24.9)
Aspirin and P2Y_12_ inhibitor	1606/1758 (91.4)	121/172 (70.3)	–	1727/1930 (89.5)
Heparin or fondaparinux	1625/1758 (92.4)	134/172 (77.9)	–	1759/1930 (91.1)
Invasive coronary angiography within 72 hours	517/907 (57.0)	36/122 (29.5)	–	553/1029 (53.7)
Invasive coronary angiography within 24 hours	91/753 (12.1)	6/39 (15.4)	–	97/792 (12.2)
Left ventricular function testing	1651/2602 (63.5)	164/441 (37.2)	1/7 (14.3)	1816/3050 (59.5)
ACEi/ARB	1299/1551 (83.8)	205/278 (73.7)	–	1504/1830 (82.2)
β Blockers	2228/2602 (85.6)	314/441 (71.2)	1/7 (14.3)	2543/3050 (83.4)
Statins	2429/2602 (93.4)	390/441 (88.4)	1/7 (14.3)	2820/3050 (92.5)
Cardiac rehabilitation	1651/2602 (63.5)	141/441 (32.0)	1/7 (14.3)	1793/3050 (58.8)

Data are number of eligible processes received divided by number of patients eligible to receive each process (%).

ACEi, ACE inhibitor; ARB, angiotensin receptor blocker.

Uptake of guideline recommended processes was 77.3% for GRS and 75.3% for standard care (OR 1.16, 95% CI 0.70 to 1.92, p=0.56) in the original UKGRIS report.^6^ In mixed logistic regression models, the effect of GRS compared with standard care on the uptake of guideline recommended care processes was greater for participants with troponin elevation compared with those without (relative OR 1.52, 95% CI 1.16 to 2.00, p<0.01 for interaction), with no effect seen in each group (with troponin elevation: OR 1.34, 95% CI 0.84 to 2.13; without troponin elevation OR 0.88, 95% CI 0.52 to 1.47).

### Time to CV composite

Troponin elevation was associated with a 13.8% increase (−2.6% to 33.0%) in the hazard of experiencing one or more composite events, with large effects of troponin elevation seen on cardiovascular death (cumulative incidence at 24 months (95% CI) of 6.6% (5.17% to 8.06%) vs 1.62% (0.26% to 3.37%)), non-fatal MI (11.2% (9.6% to 13.1%) vs 7.4% (4.3% to 11.5%)), new-onset heart failure hospitalisation (6.4% (4.9% to 7.9%) vs 3.0% (0.9% to 5.9%)) and cardiovascular readmission (46.5% (43.5% to 49.6%) vs 42.4% (36.6% to 48.6%)) (table 3).

**Table 3:** Secondary outcome measures by troponin subgroup

Outcome measure	Troponin elevated (n=2602)	Troponin not elevated (n=441)	Difference
Event	Cumulative incidence (95% CI)		HR (95% CI)
Composite (12 months)	35.5%(31.5% to 39.0%)	29.4%(24.4% to 34.2%)	1.138(0.974 to 1.330)
Composite (24 months)	48.5%(45.1% to 51.5%)	42.8%(36.7% to 49.0%)	
Cardiac death (12 months)	3.60%(2.96% to 4.26%)	1.23%(0.25% to 2.50%)	3.439(1.866 to 6.338)
Cardiac death (24 months)	6.55%(5.17% to 8.06%)	1.62%(0.26% to 3.37%)	
Non-fatal MI (12 months)	6.49%(5.58% to 7.63%)	3.44%(1.92% to 5.14%)	1.637(1.132 to 2.366)
Non-fatal MI (24 months)	11.2%(9.61% to 13.1%)	7.41%(4.29% to 11.5%)	
New-onset HF (12 months)	4.42%(3.36% to 5.60%)	1.19%(0.26% to 2.57%)	2.695(1.456 to 4.990)
New-onset HF (24 months)	6.35%(4.93% to 7.91%)	2.99%(0.92% to 5.89%)	
Cardiac readmission (12 months)	33.3%(29.9% to 36.8%)	28.6%(23.7% to 33.7%)	1.088(0.929 to 1.275)
Cardiac readmission (24 months)	46.5%(43.5% to 49.6%)	42.4%(36.6% to 48.6%)	

	Adjusted mean (95% CI)		Difference in means (95% CI)
EQ5D-5L utility (12 months)	0.686(0.660 to 0.711)	0.714(0.676 to 0.753)	−0.029(-0.062 to 0.005)

	Geometric mean (95% CI)		Ratio of geometric means (95% CI)
Duration of hospitalisation (24 months)	10.42(9.55 to 11.36)	7.84(6.95 to 8.84)	1.33(1.20 to 1.47)

For time to event outcomes, HR is the effect of elevated troponin on admission on having on or more event at any time during follow-up. For EQ5D-5L utility, 0=unconscious 1=perfect health.

EQ5D-5L, 5 Domain 5 Level version of the EuroQoL index; HF, heart failure; MI, myocardial infarction.

**Figure 2:**
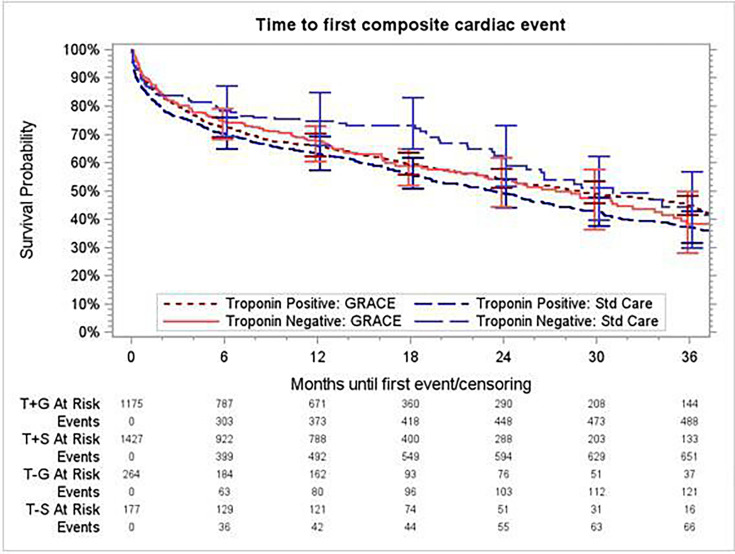
Time to first composite by randomised arm and by troponin status. GRACE, Global Registry of Acute Coronary Events; Std Care, standard care; T+G, troponin elevated in site arm randomised to GRACE; T-G, troponin not elevated in site arm randomised to GRACE; T+S, troponin elevated in site arm randomised to standard care; T-S, troponin not elevated in site arm randomised to standard care.

The time to the first composite event was not improved by the GRS (HR 0.89, 95% CI 0.70 to 1.14) or for patients without troponin elevation (1.14, 95% CI 0.79 to 1.64), and there was no interaction between troponin elevation and effect of GRS (relative HR 0.79, 95% CI 0.57 to 1.08, p = 0.14 for interaction) (figure 2). There was no significant interaction between randomisation to GRACE and troponin elevation for any of the individual components of the composite outcome (table 4).

**Table 4:** Summary table of outcome measures by troponin subgroup and by randomised arm

	Troponin elevated		Troponin not elevated		Interaction=difference (T+G C)–(T– G-C)
	GRACE	Control	GRACE	Control	
Time to event	Cumulative incidence (95% CI)				Relative HR (95%)
Composite (12 months)	33.8% (29.6% to 37.7%)	36.8% (30.6% to 42.5%)	32.2% (27.0% to 39.4%)	25.2% (15.0% to 33.6%)	0.79 (0.57 to 1.08)
Composite (24 months)	45.7% (42.2% to 48.9%)	50.8% (45.6% to 55.7%)	45.7% (38.2% to 55.5%)	38.5% (26.7% to 48.3%)	
Cardiac death (12 months)	3.48% (2.74% to 4.49%)	3.71% (2.63% to 4.60%)	1.65% (0.00% to 3.82%)	0.64% (0.00% to 2.05%)	0.52 (0.13 to 2.04)
Cardiac death (24 months)	6.74% (5.40% to 8.56%)	6.37% (3.88% to 8.54%)	1.65% (0.00% to 3.82%)	1.62% (0.00% to 5.15%)	
Non-fatal MI (12 months)	6.80% (5.88% to 7.87%)	6.22% (4.75% to 8.20%)	4.94% (2.68% to 8.36%)	1.22% (0.00% to 2.91%)	0.51 (0.22 to 1.17)
Non-fatal MI (24 months)	10.0% (8.42% to 11.9%)	12.3% (9.73% to 15.4%)	8.97% (5.09% to 14.5%)	5.11% (1.20% to 12.6%)	
New-onset HF (12 months)	3.68% (2.30% to 4.99%)	5.03% (3.48% to 6.95%)	0.40% (0.00% to 1.44%)	2.35% (0.58% to 5.39%)	0.95 (0.27 to 3.27)
New-onset HF (24 months)	5.85% (3.96% to 7.64%)	6.76% (4.76% to 9.35%)	2.53% (0.00% to 6.30%)	3.62% (0.58% to 9.87%)	
Cardiac readmission (12 months)	31.8% (27.6% to 35.6%)	34.6% (28.7% to 39.9%)	31.6% (26.4% to 39.2%)	24.1% (13.4% to 33.3%)	0.77 (0.56 to 1.07)
Cardiac readmission (24 months)	44.1% (40.3% to 47.0%)	48.6% (43.7% to 54.2%)	45.2% (37.2% to 54.5%)	38.5% (28.4% to 47.6%)	

	Baseline adjusted means				Difference in differences in means (95% CI)
EQ5D-5L utility (12 months)	0.677 (0.643 to 0.711)	0.692 (0.662 to 0.722)	0.712 (0.662 to 0.763)	0.713 (0.659 to 0.768)	−0.014 (−0.083 to 0.054)

	Geometric means (95% CI)				Relative ratio of geometric means (95% CI)
Duration of hospitalisation (24 months)	10.51 (9.36 to 11.82)	10.37 (9.34 to 11.51)	8.13 (6.94 to 9.52)	7.51 (6.35 to 8.87)	0.937 (0.768 to 1.142)

For the relative HRs, values <1 indicate that the effect of GRACE versus control on the outcome is lower among those with elevated troponin than it is for those without elevated troponin. Similarly, a relative ratio of geometric means <1 indicates that the ratio of GRACE to Control durations is smaller in elevated troponin participants than those without elevated troponin. The negative difference in difference on EQ5D-5L Utility indicates that the effect of GRACE is less for troponin positive participants than troponin negative.

EQ5D-5L, 5 Domain 5 Level version of the EuroQoL index; GRACE, Global Registry of Acute Coronary Events; HF, heart failure; MI, myocardial infarction; T+G, troponin elevated in site arm randomised to GRACE; T-G, troponin not elevated in site arm randomised to GRACE.

### Quality of life

At 12 months, those with elevated troponin had a lower adjusted mean EQ-5D-5L Utility (difference −0.029 (95% CI −0.062 to 0.005)) and had spent more days in hospital over a 24-month period (ratio of geometric means 1.33, 95% CI 1.20 to 1.47) compared with those with no troponin elevation (table 3).

Baseline adjusted EQ-5D-5L utility at 12 months was not statistically significantly different for the two trial arms among participants with troponin elevation (difference in means GRACE-Control −0.015, 95% CI −0.053 to 0.023) or without (−0.001, 95% CI −0.072 to 0.070). The duration of hospital stay within 24 months was similar for the GRS and standard care arms, respectively, among participants with or without troponin elevation (relative ratio of geometric means 0.937 (95% CI 0.768 to 1.142)) (table 4).

## Discussion

### Principal findings

In the UKGRIS randomised controlled trial of patients admitted to hospital with suspected NSTEACS, the use of the GRS improved the delivery of processes of care in those with troponin elevation relative to those without. However, this did not translate into differences in time to first of cardiovascular death, non-fatal MI, new-onset heart failure hospital admission or readmission for cardiovascular event within 24 months as captured with routine national healthcare data.

### Comparison with other studies

Troponin elevation in the context of NSTEACS has been associated with more severe coronary artery disease and intracoronary thrombus,^10 11^ risk of further cardiac events^12^ and worse prognosis.^13^ Observational studies have suggested that guideline-indicated treatment for NSTEACS is associated with improved clinical outcomes,^2 14^ and that the greatest survival benefit is derived in those with a high GRS and with elevated troponin.^15^

We found that the GRS had a greater effect on the uptake of guideline-recommended care processes for those with elevated troponin compared with those without. By contrast, in the AGRIS trial of the use of GRS led to an increase in care processes for patients without troponin elevation (OR 1.94, 95% CI 1.02 to 3.70) but not for those with troponin elevation, whether in the context of NSTEMI (OR 0.88, 95% CI 0.50 to 1.55) or STEMI (0.80, 95% CI 0.42 to 1.54).^5^ Furthermore, in AGRIS, the use of GRS led to a higher rate of early invasive coronary angiography in patients without troponin elevation (OR 4.98, 95% CI 2.71 to 9.14) compared with NSTEMI (OR 1.79, 955 CI 1.07 to 3.02) or STEMI (OR 2.01, 95% CI 0.42 to 9.82). However, for a patient without troponin elevation to be enrolled in the AGRIS trial required ECG changes or one of haemodynamic compromise, left ventricular ejection fraction <40%, diabetes mellitus or an estimated glomerular filtration rate <60 mL/min/1.73 m^2^. Compared with our less stringent inclusion criteria, the non-elevated troponin NSTEACS subgroup in the AGRIS trial may have had characteristics which would influence a clinician to treat more aggressively but are not captured in the GRS.

Though the implementation of GRS increased guideline adherence for those with elevated troponin, this did not result in a reduction in the composite outcome, even over an extended follow-up of 24 months and with a higher rate of outcomes than observed in the primary analysis. This may be because the receipt of drug treatments was high among patients with elevated troponin, likely reflecting the strong evidence base for their use.^1^ Also, in clinical guidelines, the GRS is recommended for stratifying patients for an invasive coronary strategy, rather than the other components of NSTEACS care.^1 3 4^ European guidelines recommend invasive coronary angiography within 24 hours for patients with a GRS >140, and for all patients with NSTEMI irrespective of GRS.^1^ UK National Institute of Health and Care Excellence guidelines recommend that invasive coronary angiography should be considered within 72 hours of first admission for those with NSTEACS with a predicted 6 month mortality >3.0% (equivalent to a GRS ≥89).^4^ Meta-analyses of randomised clinical trials comparing an early (<24 hours) versus a delayed invasive strategy have suggested that this may confer a survival benefit in those with troponin elevation or GRS >140,^16^ or a reduction in recurrent ischaemia and length of stay.^17^ However, the number of invasive coronary angiograms performed within 24 or 72 hours in UKGRIS patients was low (table 2), possibly because physicians were uncertain about the effectiveness of this strategy according to the evidence base despite recommendations,^17^ or that requisite catheter laboratory capacity and/or staffing resource was unavailable to meet this standard,^18^ a problem not unique to the UK.^19^ Finally, of patients with troponin elevation, 14% had a final diagnosis other than NSTEACS, and the inclusion of these patients in the analysis, where we may not expect guideline-adherent NSTEACS care to improve outcomes, may have reduced the potential to show changes in outcomes from GRS-informed care.

In total, across almost 5500 randomised patients presenting with acute coronary syndromes in AGRIS and UKGRIS, the systematic calculation and noting of the GRS at hospitals did not reduce the risk of adverse outcomes.^5 6^ Clinical care is complex, and for many patients with acute coronary syndrome, competing issues, such as comorbidities, personal preferences and social demands, may affect the ability to provide complete evidence-based care and contribute to hazard.^5^ Since the original development of the GRS two decades ago, clinical care has changed with the adoption of high sensitivity troponin assays, potent antiplatelet agents, radial access in invasive coronary angiography and improved guideline-directed medical therapy.^18 20^ For NSTEACS, clinical practice may have become ‘embedded’ in the way patients are assessed and the decisions made for intervention, and so clinicians may not prioritise the GRS results in their decisions concerning patient management in the majority of cases. While the GRS accurately describes the risk of death associated with acute coronary syndromes, its role may be to quantify the risk-benefit trade-off in specific cases, rather than to orientate management in the majority of cases presenting to hospital.

### Strengths and limitations of this study

As with the main trial, we did not adjudicate the trial endpoints collected from electronic health records data, and it is possible that there was misclassification of events.^21^ We estimated the GRS 6-month risk and applied the recommendations for treatment in hospital, which might have promoted less intensive care. This study was precipitated by a planned subgroup analysis in the primary results paper, which demonstrated that elevated troponin on admission significantly modified the effect of randomisation to GRACE in increasing uptake of care processes.^6^ However, there was an imbalance in baseline characteristics between patients with and without troponin elevation, including imbalance in factors that impact clinical decisions and outcomes but are not incorporated in the GRS (eg, diabetes mellitus). It is possible that the finding of increased uptake of care processes through the GRS in patients with troponin elevation was a type I error, and thus these findings should be considered exploratory. The majority of the trial population were men, and this precludes the potential to explore differences by sex.

A strength of this trial was the follow-up of patients using codes in national routine administrative data (including Hospital Episode Statistics and Office for National Statistics data), which has been found to be accurate and robust.^22^ This enabled the follow-up to be efficiently extended to 24 months (twice as long as reported in AGRIS) to determine that the GRS does not appear to have an impact on outcomes by troponin elevation.^5^ Data collection for longer-term outcomes can also be extracted from national databases in a cost-effective manner.

## Conclusions

In adults presenting to hospital with suspected NSTEACS, the effect of the GRS compared with standard care on uptake of recommended processes in those with troponin elevation was higher than in those without troponin elevation. However, this relative effect did not confer a greater reduction in the composite primary outcome of major cardiovascular events at 24 months.

## Supplementary material

10.1136/openhrt-2025-003213 online supplemental file 1

## Data Availability

Data are available on reasonable request.
