# Supplementary material for: Effectiveness of the GRACE risk score according to troponin elevation in patients admitted with non-ST elevation acute coronary syndrome: a post hoc analysis of the UKGRIS parallel group cluster randomised controlled trial
Source: Open Heart. 2025 Jun 26;12(1):e003213. doi: 10.1136/openhrt-2025-003213 (PMC12207166; doi:10.1136/openhrt-2025-003213)
Supplement: online supplemental file 1 [file openhrt-12-1-s001.docx]

**Supplementary Material**

Effectiveness of the GRACE risk score according to troponin elevation in patients admitted with non-ST elevation acute coronary syndrome: a post-hoc analysis of the UKGRIS parallel group cluster randomised controlled trial

Chris P Gale, Deborah D Stocken, Ramesh Nadarajah, Suleman Aktaa, Catherine Reynolds, Rachael Gilberts, David Brieger, Kathryn Carruthers, Derek P Chew, Shaun G Goodman, Catherine Fernandez, Linda D Sharples, Andrew T Yan, Keith Fox

[Supplement. Investigator list 3](#_Toc177586365)

## Supplement. Investigator list

Barking, Havering and Redbridge University Hospitals (Dr Honer Kadr)

Bradford Royal Infirmary (Dr Steven Lindsay)

Calderdale Royal Hospital/Huddersfield Royal Infirmary (Dr Jeremy Butts)

Castle Hill Hospital (Dr Richard Oliver)

Conquest Hospital (Dr Robert Gerber)

East Surrey Hospital (Dr Ansuman Saha)

Frimley Health—Wexham Park (Dr Mohammed Al-Obaidi)

Great Western Hospital (Dr Tom Hyde)

Harrogate District Hospital (Dr Bogumila Oledzka)

Imperial College Healthcare—Hammersmith Hospital (Dr Piers Clifford)

Kingston Hospital (Dr Tapesh Pakrashi)

London North West—Ealing Hospital (Dr Stuart D Rosen)

Luton and Dunstable Hospital (Dr Christopher Travill)

Northampton General Hospital (Dr Patrick Davey)

Northumbria Healthcare (Dr Iain Matthews)

Royal Oldham Hospital/North Manchester General Hospital/Fairfield General Hospital (Dr Jolanta Sobolewska) Papworth Hospital (Dr Stephen Hoole)

Pinderfields General Hospital (Dr Philip Batin)

Plymouth Hospitals NHS Trust—Derriford (Dr Girish Viswanathan)

Royal Blackburn Hospital (Dr Scot Garg)

Royal Bournemouth Hospital (Dr Terry Levy)

Royal Cornwall Hospital (Dr Sen Devadathan)

Royal Shrewsbury Hospital and Princess Royal Hospital Telford (Dr Thomas Ingram)

Royal Sussex County Hospital (Dr David Hildick-Smith)

Salisbury District Hospital (Dr Tim Wells)

Sandwell and Birmingham City Hospitals (Dr Vinoda Sharma)

Sherwood Forest Hospitals (Dr Sukhbinder Bassi)

South Tyneside and Sunderland NHS Foundation Trust (Dr Shahid Junejo)

Torbay Hospital (Dr Philip Keeling)

University Hospital Lewisham (Dr Khaled Alfakih)

University Hospitals of Morecambe Bay NHS Trust (Adrian Brodison)

University Hospital of North Tees (Justin Carter)

Victoria Hospital, Blackpool (Dr Ranjit Moore)

West Middlesex University Hospital (Dr Francesco LoMonaco)

Whipps Cross (Dr Anil Kumar Taneja)

Worcestershire Royal Hospital (Dr Helen Routledge)

Wycombe General Hospital (Dr Piers Clifford)

Yeovil District Hospital (Dr Andrew Broadley)

York Hospital (Dr Chris Hayes).
